# Minding the gap. Drug-related problems among breastfeeding women

**DOI:** 10.3389/fphar.2025.1542269

**Published:** 2025-03-13

**Authors:** Karolina Morze, Edyta Szałek, Magdalena Waszyk-Nowaczyk

**Affiliations:** ^1^ Department of Clinical Pharmacy and Biopharmacy, Poznan University of Medical Sciences, Poznan, Poland; ^2^ Pharmacy Practice and Pharmaceutical Care Division, Department of Pharmaceutical Technology, Poznan University of Medical Sciences, Poznan, Poland

**Keywords:** drug-related problems, breastfeeding, pharmaceutical care, polypharmacy, lactation, medication-related problems

## Abstract

**Introduction:**

Drug-related problems (DRPs) are a significant concern in many patient populations, including breastfeeding women. This study aimed to identify and characterize those problems in a group of breastfeeding women seeking specialized pharmaceutical care.

**Materials and methods:**

A prospective observational study was conducted among women who registered for a pharmacist’s online consultation regarding medication safety in lactation. 200 patients were enrolled. Patient medical history, medication use, breastfeeding practices, and DRPs were assessed. DRPs were classified using the Pharmaceutical Care Network Europe Association (PCNE) classification system. Causality assessment for adverse events was performed using the Naranjo algorithm and the Liverpool Causality Assessment Tool (LCAT).

**Results:**

This study found a high prevalence of DRPs among 190 out of 200 breastfeeding women. Of these, 27 experienced potential DRPs, and 163 manifested actual DRPs. A total of 218 DRPs were identified, with ineffective therapy being the most frequent (63.3%, n = 138). Among all identified causes (n = 265), the most common were patient-related factors (47.5%, n = 126) and dispensing-related issues, particularly regarding the information provided to patients about medication safety during lactation. Pharmacist interventions were accepted by 79.5% (n = 151) of patients, with 70% (n = 133) of DRPs successfully resolved.

**Conclusion:**

This study highlights the significant burden of DRPs among breastfeeding women and the potential for medical professionals to improve patient outcomes through evidence-based interventions. Future research should focus on developing evidence-based guidelines for medication use during lactation and improving healthcare provider education to optimize maternal and infant health.

## 1 Introduction

Drug-related problems (DRPs) are defined as events or circumstances involving drug therapy that actually or potentially interfere with the desired health outcome (Pharmaceutical Care Network Europe Association, 2020). DRPs are increasingly recognized as a significant contributor to medication-related harm and suboptimal patient outcomes (Cipolle et al., 2012; WHO, 2024). They can occur at any stage of the medication use process, and lead to reduced overall quality of life and increased morbidity, mortality, and healthcare costs (Sheleme et al., 2021; Garin et al., 2021; Wohlgemuth et al., 2020; Prasad et al., 2024; WHO, 2024).

Identifying and addressing DRPs is a crucial role for healthcare providers, particularly pharmacists. By proactively identifying risk factors, conducting medication therapy reviews, and implementing appropriate interventions, it is possible to mitigate the impact of DRPs and improve patient outcomes (Greeshma et al., 2018; Ayele and Tesfaye, 2021).

Patient-related factors, such as polypharmacy, lack of knowledge, comorbidities, and advanced age, have been well-documented as risk factors for DRPs (Sell and Schaefer, 2020; Prasad et al., 2024). However, a significant gap persists in our understanding of DRPs in specific populations, including breastfeeding women.

Breastfeeding is widely recognized as a cornerstone of infant and maternal health, offering numerous long-term benefits (Brown et al., 2014; Pérez-Escamilla et al., 2023; Louis-Jacques and Stuebe, 2020). Despite these benefits, many women struggle to achieve the recommended duration of breastfeeding, potentially compromising the health of both mother and child. Approximately 10% of mothers who discontinue breastfeeding before the recommended 6 months (World Health Organization) cite medical reasons as a primary factor influencing their decision (Brown et al., 2014; Inano et al., 2021).

Our previous research (Morze et al., 2024a) highlighted therapy effectiveness as a common DRP among breastfeeding women with depressive spectrum disorders. In this study, we aim to expand upon these findings by examining a broader population of breastfeeding women with various medical conditions. Specifically, we will investigate the prevalence of DRPs in this population, identify common problems, and explore the possible ways to solve them.

This manuscript primarily uses the term ‘breastfeeding’ to encompass all methods of feeding an infant with human milk, including chestfeeding by transgender, non-binary, and other gender-diverse parents. We acknowledge that the term ‘breastfeeding’ may have varying connotations and that the act of feeding an infant with human milk can be achieved through different methods. This research aims to contribute to a broader understanding of the benefits and challenges of feeding infants with human milk.

## 2 Materials and methods

**FIGURE 1:**
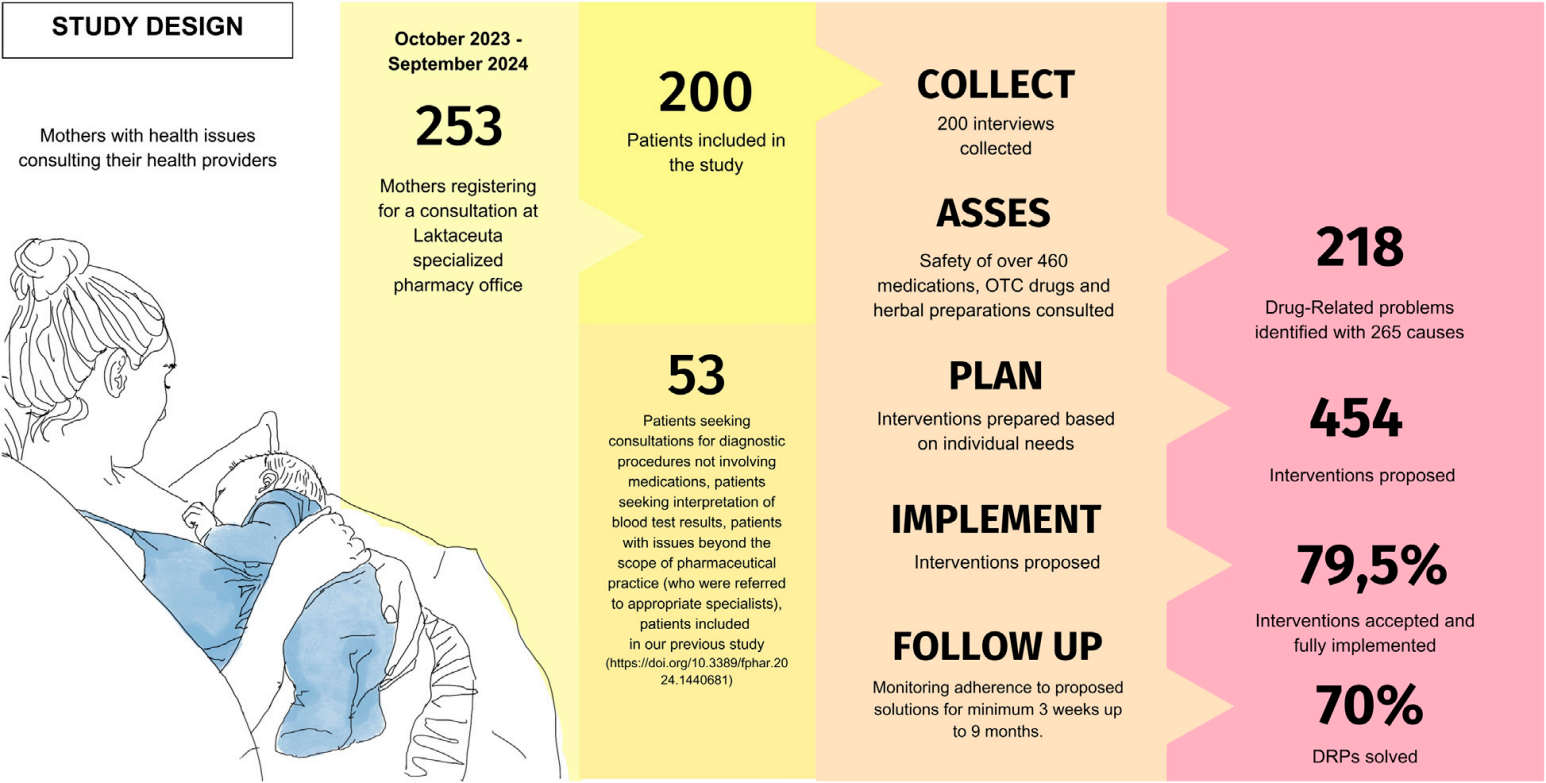
Study design.

This is a prospective observational study conducted among breastfeeding women who registered for a pharmacist’s consultation between September 2023 and October 2024. Figure 1 presents the study design.

### 2.1 Study setting

This study was conducted at Laktaceuta specialized pharmacy office in Poznań providing lactation support and medication consultations. This private center offers online services, including medication safety assessments, over-the-counter drug recommendations, treatment plan support, and DRP identification and resolution. Patients across Poland accessed the online platform after free registration, with consultations and other services subject to a fee.

### 2.2 Participants

From 23 September 2023 to 30 October 2024, a total of 253 patients from different locations in Poland registered for consultations.

Exclusion criteria for this study included patients seeking consultations for diagnostic procedures not involving medications, those with issues beyond the scope of pharmaceutical practice (who were referred to appropriate specialists), and those who had participated in our previous study regarding DRPs.

A total of 200 patients were included in the study. The patients were either breastfeeding or pregnant and planning to breastfeed.

### 2.3 Study design

This study employed an analysis of data collected during pharmacist consultations. The consultation workflow adhered to a five-step process adapted from the Joint Commission of Pharmacy Practitioners Pharmacists’ Patient Care Process standards (Joint Commission of Pharmacy Practitioners, 2014).

#### 2.3.1 Collecting information

Interviews using the MILC questionnaire (Morze et al., 2024a) facilitated efficient exploration of patient needs, current medications and supplements, maternal and infant health status, breastfeeding practices, lifestyle factors and potential DRPs.

In-Depth Interviews: When necessary, additional in-depth interviews were conducted to gather more detailed information about specific concerns, such as adverse events (AEs).

Clinical Records Review: Whenever possible, access to patients’ medical records was obtained to review past medical history, physical examination findings, and physician visit summaries. This data supplemented the information obtained through interviews.

#### 2.3.2 Assessing

A comprehensive evaluation was conducted to identify and categorize potential and existing DRPs based on the Pharmaceutical Care Network Europe (PCNE) Classification System version 9.1 (Pharmaceutical Care Network Europe Association, 2020).

Literature & Database Review: For medication safety assessments in the context of breastfeeding, resources such as LactMed (Drugs and Lactation Database, 2006), E-Lactancia (E-Lactancia.org), Safety Score (Uguz, 2021), PubMed searches, drug Summary of Product Characteristics (SmPCs), and “Medications and Mothers Milk” Manual 2023 (Hale and Krutsch, 2023; Hale and Krutsch, 2024) were utilized.

#### 2.3.3 Planning

Interdisciplinary collaboration with specialists from other healthcare fields was implemented when necessary to ensure optimal patient care. Based on identified DRPs and patients’ needs, evidence-based interventions were planned.

#### 2.3.4 Implementing

Patient consultations were conducted to ensure concordance with treatment recommendations.

Counseling was based on established frameworks, including a modified version of the Calgary-Cambridge Guide (Greenhill et al., 2011) and the Joint Commission of Pharmacy Practitioners’ Patient Care Process (Joint Commission of Pharmacy Practitioners, 2014). We utilized a tailored counseling tool specifically developed for medication safety in lactation (Morze et al., 2024b). Motivational dialogue techniques were also incorporated into patient communication.

Patient education included patient-specific written instructions, outlining medication risks and benefits during lactation, along with specific safety assessment recommendations. Professional-facing fact sheets were provided to healthcare providers on patients’ medications, covering key safety information, breastfeeding considerations, and monitoring recommendations, when needed. In some cases, brief video consultations were conducted to address individual concerns and provide personalized counseling. Patients were provided with a list of local lactation support specialists upon request. When deemed relevant, patients were directed to educational resources from reputable national societies and support centers on topics such as healthy lifestyle, treatment adherence, and disease management.

#### 2.3.5 Follow-up

Follow-up consultations were conducted to monitor patient outcomes, adjust interventions as needed, and ensure continued breastfeeding success.

Consistent with the latest WHO guidance (WHO, 2024), the study aimed to identify not only existing DRPs but also potential ones.

### 2.4 Adverse event management

Maternal descriptions were the primary source for identifying AEs. To address potential bias associated with subjective reporting, a comprehensive evaluation was conducted upon identifying a suspected AE. This evaluation considered factors related to the mother, the lactation process, and the infant, aiming to establish a clear medication-effect link.

Standardized MedDRA codes (version 27.1) were used to ensure data consistency and clarity in reporting AEs. The Common Terminology Criteria for Adverse Events (CTCAE) scale version 5.0 guided the severity grading of AEs. Causality assessment of DRPs employed two established tools: the Naranjo scale (Naranjo et al., 1981) and the Liverpool Causality Assessment Tool (LCAT) (Gallagher et al., 2011).

These tools facilitated a standardized approach to evaluating the likelihood that the identified DRPs were caused by the medications being taken by the breastfeeding mothers.

Health issues in this study were classified according to the International Classification of Diseases (ICD-11) when applicable.

## 3 Results

### 3.1 Participants

The study population comprised 200 breastfeeding mothers and their babies. The most prevalent health conditions among these mothers were mental, behavioral, or neurodevelopmental disorders (such as depression, anxiety, neurosis, insomnia, ADHD, bipolar disease and autism), followed by thyroid disorders (hyperthyroidism and Graves disease), digestive system diseases (gastritis, ulcerative colitis, Crohn’s disease, pancreatitis) and nervous system disorders (multiple sclerosis, epilepsy, migraine, sciatic nerve disorders). Table 1 summarizes patient data.

**TABLE 1:** Maternal and infant data based on the MILC Questionnaire including lactation (S, arithmetic mean; SD, standard deviation; DRP, drug-related problem; BMI, body mass index).

Mothers		n = 200	
Anthropometric parameters		S	SD
Weight		67.2 kg	13.5
Height		166.9 cm	5.21
BMI		24.16	4.61
Health issues		n	**%**
Mental, behavioural or neurodevelopmental disorders		38	19
Disorders of the thyroid gland or thyroid hormones system		21	10.5
Diseases of the digestive system		19	9.5
Diseases of the nervous system		17	8.5
Infectious agents		13	6.5
Diseases of the genitourinary system		13	6.5
Surgery		11	5.5
Other		11	5.5
Diseases of the musculoskeletal system or connective tissue		10	5
Certain infectious or parasitic diseases		9	4.5
Diagnostic procedures		8	4
Diseases of the circulatory system		6	3
Diseases of the respiratory system		6	3
Diseases of the ear or mastoid process		5	2.5
Diseases of the visual system		5	2.5
Diseases of the immune system		4	2
Diseases of the urinary system		4	2
Total		200	100

Children			
Anthropometric parameters		S	SD
Age		9.72 months	9.02
Weight		8,211 g	2,847
How baby is fed		n	%
Exclusive breastfeeding		73	36.5
Exclusive breastfeeding with solids		94	47
Exclusive breastfeeding and expressed milk		1	0.5
Expressed milk		3	1.5
Expressed milk with solids		2	1
Mixed breastfeeding/expressed breastmilk and formula feeding		10	5
Formula feeding		12	6
Preparing to breastfeed		3	1.5
Unknown		2	1
Total		200	100

Lactation			
Breastmilk share in child’s diet		n	**%**
100%		92	46
75%		37	1,5
50%		20	10
25%		14	7
Less than 25%		24	12
No data		13	6.5
Total		200	100

In addition to the most common health conditions, a small number of patients (n = 8) sought consultations regarding medication safety during lactation for diagnostic procedures and surgical procedures (n = 11).

Furthermore, 11 cases involved rare conditions that did not align with the most common categories in this study.

The majority of mothers (96%, n = 192) have been prescribed medications within the recommended dosing range. However, a minority experienced dosing discrepancies: 1.5% (n = 3) received doses that were too high, 1.5% (n = 3) received doses that were too low.

Polypharmacy occurred in 30.5% (n = 61) of the mothers - they were taking multiple medications, including prescription drugs, over-the-counter medications, dietary supplements, and herbal preparations. No clinically significant interactions were detected.

Among the study population, 14 mothers were pregnant. Of these, 11 were concurrently breastfeeding, while three were primiparous women with chronic conditions who were preparing to initiate breastfeeding. All these women successfully initiated breastfeeding after childbirth.

The average age of the infants was approximately 9.7 months. The majority of infants (92%, n = 167) were primarily breastfed, either exclusively or in combination with solid foods. A smaller proportion (6%, n = 12) received a combination of breast milk and formula. Twelve mothers (6%) abruptly stopped breastfeeding and introduced formula due to medical advice to start pharmacological treatment.

Approximately 27% (n = 55) of infants had identified health issues not related to maternal medications, such as anemia, reflux, allergy, colic, atopic dermatitis, infection.

### 3.2 Drug-related problems

During the interviews, a total of 218 DRPs were identified among 190 patients. Of these patients, 144 experienced a single DRP, 19 experienced multiple DRPs, 27 were at risk of experiencing DRPs (potential DRPs), and 10 did not experience any DRPs.

The most frequent DRP identified was “Effect of drug treatment not optimal”, accounting for 63.3% of all DRPs (n = 138). Other common DRPs included untreated symptoms or indications (8%, n = 16), potential adverse drug reactions (4.5%, n = 9), and unnecessary drug treatment (1%, n = 2). Additionally, 26.5% of DRPs (n = 53) involved unnecessary interventions impacting breastfeeding, a significant finding that warrants further investigation.

In these cases, prescribers recommended interventions such as complete breastfeeding cessation, significant reduction in breastfeeding sessions, extended pauses between medication dosing and breastfeeding, formula supplementation, and milk pumping and discarding. These interventions were not supported by current evidence-based data and may have had negative impacts on lactation, maternal, and infant wellbeing and wide-ranging health outcomes. Consequently, we classified these instances as DRPs (that would fall in the “Other” category in PCNE DRP 9.1 classification). It is worth noting that the largest share of this type of DRP was observed in cases involving surgical procedures and diagnostic procedures.

No cases of “lack of medication effectiveness despite correct use” were identified among the study population.

Table 2 presents the distribution of manifested and potential DRPs among 190 patients, categorized by specific health conditions.

**TABLE 2:** Distribution of manifested and potential Drug-Related Problems (DRPs) among 190 patients.

Patients n = 190							
DRP code and problem	1.2	1.3	2.1	3.1	3.2	Total	
	Effect of drug treatment not optimal	Untreated symptoms or indication	Adverse drug event (possibly) occurring	Unnecessary drug treatment	Other: Unnecessary interventions in breastfeeding^a^		
Maternal health problems	No of DRPs	No of DRPs	No of DRPs	No of DRPs	No of DRPs	No of DRPs	%
Certain infectious or parasitic diseases	9	—	—	—	—	9	4.1
Diagnostic procedures^a^	—	—	—	—	6	6	2.8
Diseases of the circulatory system	3	1	—	—	2	6	2.8
Diseases of the digestive system	14	2	1	1	5	23	10.6
Diseases of the ear or mastoid process	5	—	—	—	1	6	2.8
Diseases of the genitourinary system	12	—	—	—	1	13	6.0
Diseases of the immune system	3	—	—	—	1	4	1.8
Diseases of the musculoskeletal system or connective tissue	6	—	1	—	4	11	5.0
Diseases of the nervous system	6	3	2	—	6	17	7.8
Diseases of the respiratory system	6	—	—	—	—	6	2.8
Diseases of the urinary system	4	—	—	—	—	4	1.8
Diseases of the visual system	3	1			2	6	2.8
Endocrine, nutritional or metabolic diseases	18	4			3	25	11.5
Infectious Agent	9	3	—	—	2	14	6.4
Mental, behavioural or neurodevelopmental disorders	31	1	4	1	8	45	20.6
Other^a^	8	1	1	—	3	13	6.0
Surgery^a^	1	—	—	—	9	10	4.6
Total No of DRPs	138	16	9	2	53	218	100
%	63.3	7.3	4.1	0.9	24.3	100	

Description: Maternal health conditions were classified using the highest level of the International Classification of Diseases, 11th Revision (ICD-11). DRPs were classified using the Pharmaceutical Care Network Europe Association (PCNE) classification system, version 9.1.

^a^ Note: Diagnostic Procedures and Surgery categories, while not explicitly included in ICD-11, were added to represent the full study population. The “Other” category encompasses patients with rare conditions.

A total of 265 potential causes for DRPs were identified. The most frequent cause was patient-related factors, accounting for 47.5% (n = 126) of causes, primarily due to non-adherence (patients took lower doses or did not take prescribed medications at all). Dispensing-related problems were the second most common, representing 29.8% (n = 79) of causes. These issues were largely attributed to inaccurate information provided to patients regarding medication safety during lactation at various stages of care. Drug selection problems accounted for 12.5% (n = 33) of causes, with the most common issue being the absence or incompleteness of treatment despite indications. Other contributing factors included dose selection (3%, n = 8), treatment duration (1.9%, n = 3), and various unspecified causes (5.3%, n = 14).

### 3.3 Adverse events

A total of 16 potential AEs were identified among the 200 patients (8%). Of these, 7 AEs affected both the mothers and lactation (3.5%), while 9 (4.5%) AEs affected the infants.

One case of definite causality between an adverse event and medication was identified. A mother taking methylphenidate for ADHD and levothyroxine for hypothyroidism experienced a significant increase in anxiety (CTCAE grade 2) after initiating methylphenidate. This issue was discussed with the prescribing psychiatrist, leading to methylphenidate dosage adjustments.

Five maternal cases were classified as probable adverse drug reactions based on the Liverpool Causality Assessment Tool (LCAT), but the causality result was not consistent with the Naranjo algorithm.

Table 3 details seven cases of AEs reported by mothers. For each case, the table lists all medications the mother was taking at the time of the AE (all doses taken at the moment the AE occurred were within recommended manufacturers regimen). Additionally, the table includes a description of the AE using MedDRA terms (version 27.1) and the results of causality assessments using both the Naranjo algorithm and the LCAT tool. The severity of each AE was graded using the CTCAE scale.

**TABLE 3:** Adverse events among mothers, including the effects on lactation.

Patient ID	Maternal medication (s)	MedDra term v27.1	Naranjo score	LCAT	CTCAE v5.0
1986	**Sertraline**, levothyroxine	Somnolence	6	Probable	1
1814	**Sertraline**, etamsilat, rutoside and hesperidin herbal OTC drug	*Postartum* bleeding	4	Probable	2
1631b2	Rifampicin, **neomycin**, mebeverine, lisinopril, spironolactone	Vomiting	7	Probable	1
1855	**Methylphenidate**, levothyroxine	Anxiety	10	Definite	2
1975	**Estradiol**, acetylsalicylic acid low dose, **progesterone**, filgrastim, enoxaparin^a^	Lactation decreased	2	Probable	1
1956	**Thiamazole**	Lactation decreased	2	Possible	1
1610	**Prednisone**, esomeprazole, **progesterone** contraceptive, vitamin B low dose supplement	Lactation decreased	5	Probable	1

^a^ Patient was pregnant, therefore the adverse event concerning lactation might have been connected to pregnancy.

Bold indicates medications that the mothers suspected were the cause of the AE.

Description: Adverse Events (AEs) among infants were classified using MedDRA, terms (version 27.1). Causality assessment was conducted using both the Naranjo algorithm and the Liverpool Causality Assessment Tool (LCAT). The Naranjo algorithm categorizes causality as follows: 1–4 points, possible; 5–8 points, probable; and 9 points and above, definite. The severity of AEs was graded according to the Common Terminology Criteria for Adverse Events (CTCAE) version 5, with Grade 1 representing the mildest severity and Grade 5 representing the most severe.

Among infants, nine potential AEs were reported (4.5%). Based on the LCAT tool, five of these were classified as probable, two as possible, and two as unlikely to be related to maternal medications. However, the Naranjo algorithm did not support a causal link between any of these AEs and maternal medication. Therefore, it is uncertain whether maternal medications contributed to these adverse events at all.

Table 4 details nine cases of AEs reported among infants. For each case, the table lists all medications the mother was taking at the time of the AE (all doses taken at the moment the AE occurred were within recommended manufacturers regimen), the child’s age, and the proportion of breast milk in the child’s diet. Additionally, the table includes a description of the AE using MedDRA terms (version 27.1) and the results of causality assessments using both the Naranjo algorithm and the LCAT tool. The severity of each AE was graded using the CTCAE scale.

**TABLE 4:** AEs among infants.

ID	Maternal medication(s)	Child’s age (months)	Breastmilk share in child’s diet (%)	MedDra term v27.1	Naranjo score	LCAT	CTCAE v5.0
1859	Topiramate, methylprednisolone, hydroxychloroquine, enoxaparin	0.1	25	Vomiting	3	Probable	1
1952	Certolizumab, lamotrigine, quetiapine, iron supplement, B12, vitamin D, magnesium-potassium supplement	0.5	75	Irritability	0	Probable	1
1873	Doxylamine, levothyroxine	28	25	Somnolence	2	Probable	1
1733	Tropicamide, fluorescein, enoxaparin, insulin	10	100	Low grade fever	2	Unlikely	1
1619b^a^	Paroxetine^a^, prenatal vitamins	0	50	Somnolence	3	Probable	2
1896	Amoxicillin, paracetamol + codeine	11	Not known	Loose stools	2	Probable	1
1801	Glatiramer acetate	0	50	Hypoglycemia	1	Possible	1
1986	Sertraline, levothyroxine	2.5	100	Rash	2	Possible	2
1714	Sertraline	9	50	Decreased appetite, difficulty sleeping	2	Unlikely	1

^a^ In this case the most likely reason for AE, was transplacental exposure, mother was taking paroxetine from the II, trimester of pregnancy.

Description: Adverse Events (AEs) among infants were classified using MedDRA terms (version 27.1). Causality assessment was conducted using both the Naranjo algorithm and the Liverpool Causality Assessment Tool (LCAT). The Naranjo algorithm categorizes causality as follows: 1–4 points, possible; 5–8 points, probable; and 9 points and above, definite. The severity of AEs was graded according to the Common Terminology Criteria for Adverse Events (CTCAE) version 5, with Grade 1 representing the mildest severity and Grade 5 representing the most severe.

### 3.4 Interventions, acceptance and outcomes

A total of 454 interventions were implemented for 190 patients with identified DRPs. The most common interventions included patient counseling (39.6%, n = 180), recommendations to initiate prescribed medications (28.6%, n = 130), and adjustments to breastfeeding practices based on current evidence (11.7%, n = 53). Other interventions involved referrals to prescribers (7.7%, n = 35), modifications to medication instructions (6.6%, n = 30), and various other strategies such as consultations with prescribers and dosage adjustments.

Patient adherence to the proposed interventions was high, with 79.5% (n = 151) of patients fully implementing the recommended actions. A small proportion of patients agreed upon recommended interventions, but partially implemented (2.6%, n = 5) or did not implement (3.2%, n = 6) the interventions. In 2.1% of cases (n = 4) there was agreement, but the implementation status was unknown. Due to a lack of patient contact both the acceptance and implementation remained unknown for 11.1% of cases (n = 21). Additionally, 1.5% of patients (n = 3) did not accept the proposed interventions.

The overall resolution rate for DRPs was 70% (n = 133), while the problem status remained unknown for a total of 25.3% (n = 48). In 4.8% of cases (n = 9), the problem was partially resolved or not resolved.

**FIGURE 2:**
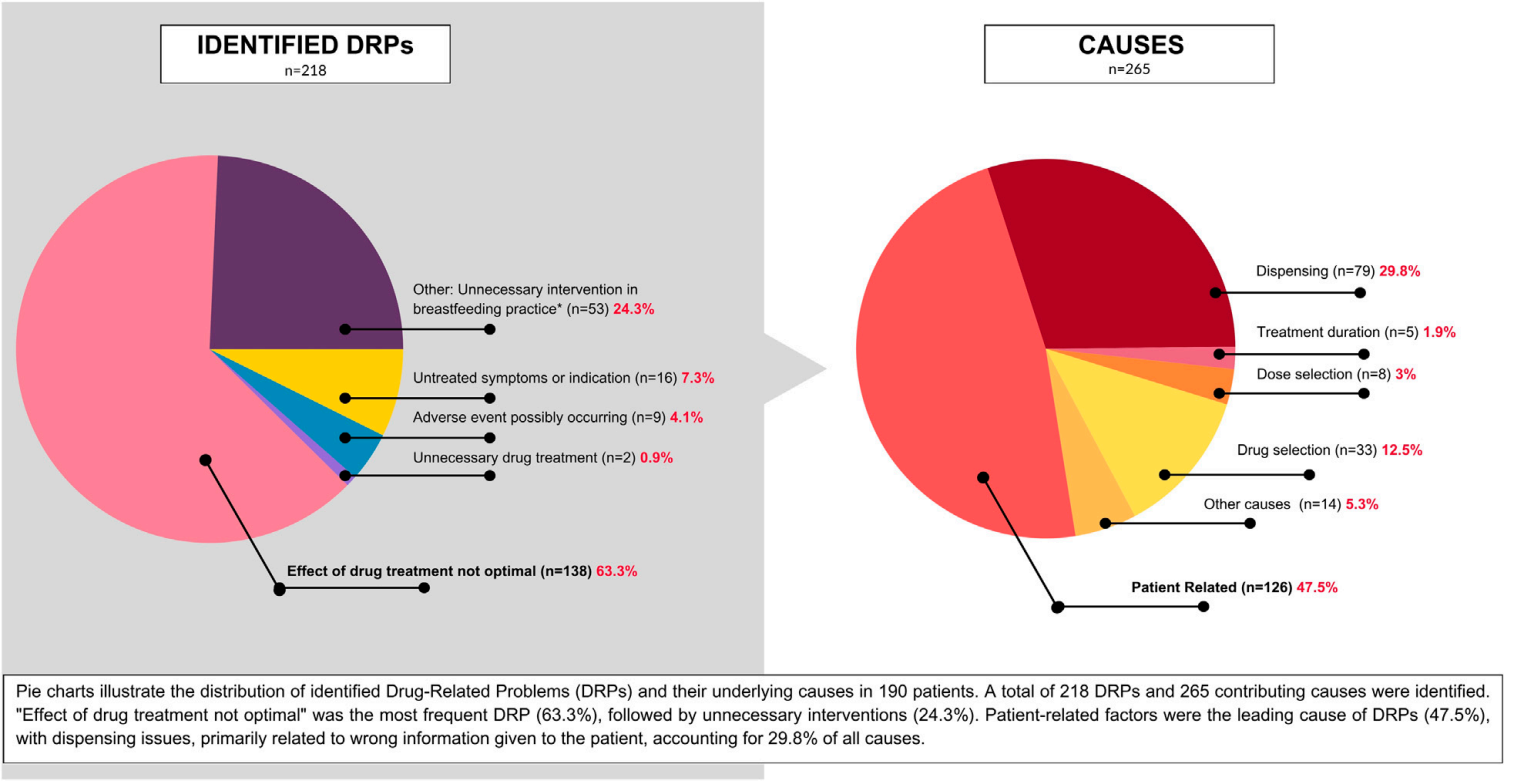
Distribution of the incidence of DRPs in the group of 190 patients and identified causes. A total of 218 DRPs and 265 causes were identified among 190 patients.

**FIGURE 3:**
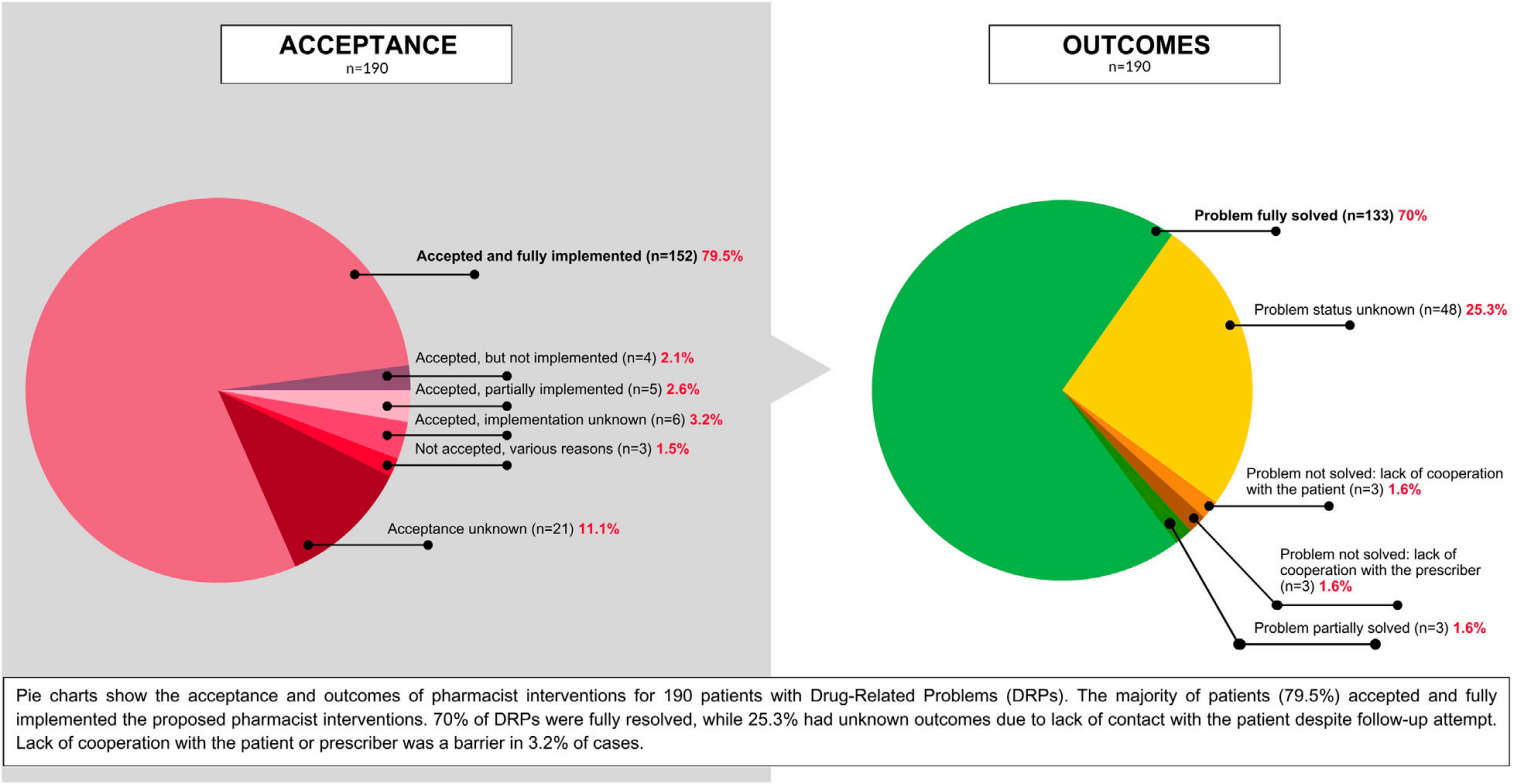
Pie charts showing the acceptance level of the proposed pharmacist interventions for patients with DRPs, and the outcomes of pharmaceutical consultations.

Figures 2, 3 represent the distribution of DRPs, their identified causes, acceptance level and final outcome of the consultations among the patients.

## 4 Discussion

This study highlights the significant prevalence of DRPs among breastfeeding women with diverse health conditions. The findings underscore the critical need for DRP screening to optimize medication therapy and minimize potential adverse outcomes to both mothers and infants.

The most prevalent DRP identified in our study group was ineffective drug treatment, indicating that many women were not achieving optimal therapeutic outcomes. This may be attributed to various factors, including patient non-adherence and lack of up-to-date knowledge of medical personnel. The high frequency of dispensing-related problems emphasizes the importance of accurate and evidence-based information dissemination to healthcare providers and patients regarding medication safety during lactation.

This study confirms the findings of our previous, smaller study (Morze et al., 2024a), which also identified “effect of drug treatment not optimal” as the most frequent DRP among breastfeeding women with depressive spectrum disorders. By expanding the study population to include a broader range of health conditions, this study further emphasizes the prevalence of this DRP. Moreover, the underlying causes of ineffective drug treatment remain consistent with those identified in the previous study.

Our findings align with some observations in pregnant populations, although differences exist depending on the study setting (hospital vs community). Limited data in pregnant populations have demonstrated a high prevalence of DRPs. A Norwegian study found that 42% of women in maternity wards experienced at least one DRP, with the “need for additional drug” being the most frequent (Smedberg et al., 2016). Other studies have identified therapeutic ineffectiveness and adverse drug reactions as common DRPs in pregnant women, particularly in those with conditions like hypertension and gestational diabetes mellitus (Vale Bezerra et al., 2023). Adherence issues, such as delayed treatment initiation and non-adherence to prescribed regimens, have been observed in pregnant women in community settings (de Korte et al., 2023), and these findings align with our observations in lactating women.

Data on DRPs in vulnerable populations, including lactating women, are limited and often difficult to compare due to variations in study settings, patient characteristics, and research methodologies. While some similarities may exist between DRPs in lactating and pregnant women, given their shared vulnerability and the potential impact of medications on their offspring, significant physiological differences necessitate separate assessments.

Given the established risk factors for DRPs, such as lack of knowledge, polypharmacy, comorbidities, and advanced age (Sell and Schaefer, 2020; Prasad et al., 2024; WHO, 2024), we propose that breastfeeding may also be considered a risk factor, particularly for ineffective treatment.

This study identified a 30% prevalence of polypharmacy among breastfeeding women. While there have been studies among other valuable populations concerning polypharmacy, like pregnant women (Subramanian et al., 2023), further research is needed to explore the optimal management of polypharmacy in the breastfeeding population.

A small number of potential AEs were identified in our population. It is noteworthy that in most cases where maternal AEs were identified, no corresponding AEs were observed in the infants, with the exception of one case. The majority of AEs were probably not linked to maternal medication. This highlights the challenge of accurately attributing AEs to specific medications, particularly in complex clinical scenarios. Discrepancies between causality assessments using the Naranjo algorithm and the LCAT have raised concerns. These discrepancies may arise from differences in methodologies, with each tool employing distinct criteria and scoring systems, potentially leading to varying interpretations of the same evidence. For example, the Naranjo algorithm, unlike the LCAT, incorporates a criterion assessing the reappearance of the adverse event upon placebo administration, a factor that would be of more use in clinical trial research. Furthermore, inherent subjectivity within the assessment process can contribute to variability.

Further research is warranted to investigate the factors contributing to these discrepancies. It should include comparative studies of different causality assessment tools in larger cohorts to identify consistent patterns of disagreement. Additionally, assessing the inter-rater reliability of different assessors using the same causality assessment tool is needed to understand the impact of subjectivity. Exploring alternative methodologies for assessing causality of AEs may offer valuable insights.

The study also revealed a high rate of unnecessary interventions recommended for breastfeeding women, including complete breastfeeding cessation and significant reductions in breastfeeding frequency. These interventions, often not supported by evidence-based data, can have detrimental effects on breastfeeding outcomes and infant health. We believe that pharmacists can play a crucial role in educating healthcare providers and patients about evidence-based recommendations for medication use during lactation, minimizing the risk of unnecessary interventions, but such role is not limited to pharmacists only. Preventing, screening, and addressing DRPs could take place at any point of medical care by any trained personnel.

This manuscript also highlights the need for enhanced education and training programs for healthcare providers on the safe use of medications during lactation. To address this need, future research should focus on developing effective educational strategies to improve prescriber knowledge and awareness in this field, as well as creating user-friendly decision-support tools to assist in making informed decisions regarding medication and breastfeeding.

Furthermore, the development and implementation of clinical guidelines can significantly improve the decision-making process for healthcare providers. These guidelines should prioritize the unique needs of the breastfeeding dyad. A multi-faceted approach to guideline development is crucial, encompassing systematic reviews and meta-analyses, incorporation of expert consensus from international specialists, pilot testing, and continuous review and updating.

The high rate of patient acceptance and implementation of pharmacist-recommended interventions underscores the value of pharmaceutical care in optimizing medication therapy for breastfeeding women. However, further efforts are needed to improve patient adherence to recommended interventions, particularly in cases requiring lifestyle modifications or complex medication regimens.

While this study provides valuable insights into the prevalence and nature of DRPs among breastfeeding women, it is important to acknowledge the following limitations.

- Limited Sample Size: The sample size, while significant, may not be fully representative of the broader population of breastfeeding women. A larger sample size could provide more robust statistical power and enable comparisons of intervention effectiveness across different DRP categories.
- Self-Reported Data: Reliance on patient self-reported information for AEs and medication adherence may introduce bias.
- Single-Center Study: The findings may not be generalizable to other healthcare settings or regions with different healthcare systems and patient populations.

## 5 Conclusion

This study highlights the significant prevalence of drug-related problems among breastfeeding women, particularly ineffective treatment and unnecessary interventions in breastfeeding practice. By addressing medication-related concerns and providing accurate information, pharmacists can optimize pharmacotherapy, minimize adverse events, and support successful breastfeeding to help patients achieve their lactation goals.

## Data Availability

The original contributions presented in the study are included in the article/supplementary material, further inquiries can be directed to the corresponding author.
